# Coagulation Changes during Central Hypovolemia across Seasons

**DOI:** 10.3390/jcm9113461

**Published:** 2020-10-27

**Authors:** Nandu Goswami, Alexander Andreas Taucher, Bianca Brix, Andreas Roessler, Martin Koestenberger, Gilbert Reibnegger, Gerhard Cvirn

**Affiliations:** 1 Physiology Division, Otto Loewi Research Center, Medical University of Graz, 8036 Graz, Austria; andreas.taucher@stud.medunigraz.at (A.A.T.); bianca.brix@medunigraz.at (B.B.); andreas.roessler@medunigraz.at (A.R.); 2 Department of Pediatric Cardiology, Medical University of Graz, 8036 Graz, Austria; martin.koestenberger@medunigraz.at; 3 Physiological Chemistry Division, Otto Loewi Research Center, Medical University of Graz, 8036 Graz, Austria; gilbert.reibnegger@medunigraz.at (G.R.); gerhard.cvirn@medunigraz.at (G.C.)

**Keywords:** seasons, coagulation, LBNP, thrombelastometry, thrombin generation, trhombogram

## Abstract

Lower body negative pressure (LBNP) application simulates hemorrhage. We investigated how seasons affect coagulation values at rest and during LBNP. Healthy participants were tested in cold (November–April) and warm (May–October) months. Following a 30-min supine period, LBNP was started at −10 mmHg and increased by −10 mmHg every five minutes until a maximum of −40 mmHg. Recovery was for 10 min. Blood was collected at baseline, end of LBNP, and end of recovery. Hemostatic profiling included standard coagulation tests, calibrated automated thrombogram, thrombelastometry, impedance aggregometry, and thrombin formation markers. Seven men (25.0 ± 3.6 years, 79.7 ± 7.8 kg weight, 182.4 ± 3.3 cm height, and 23.8 ± 2.3 kg/m^2^ BMI) and six women (25.0 ± 2.4 years, 61.0 ± 8.4 kg weight, 167 ± 4.7 cm height, and 21.8 ± 2.4 kg/m^2^ BMI) participated. Baseline levels of prothrombin (FII), tissue factor (TF) and markers for thrombin generation F1+2 and the thrombin/antithrombin complex (TAT) were higher during summer. Factor VIII, prothrombin fragment 1+2 (F1+2), TAT and the coagulation time showed significant increases during LBNP in both seasons. Some calibrated automated thrombography variables (Calibrated automated thrombography (CAT): lag, time to peak (ttPeak), peak) shifted in a procoagulant direction during LBNP in summer. Red blood cell counts (RBC), hemoglobin and white blood cell counts (WBC) decreased during LBNP. LBNP application reduced prothrombin time in winter and activated partial thromboplastin time in summer. Greater levels of FII, TF, F1+2, and TAT—a more pronounced LBNP-induced procoagulative effect, especially in CAT parameters (lag time (LT), Peak, ttPeak, Velindex)—were seen in summer. These results could have substantial medical implications.

## 1. Introduction

Lower body negative pressure (LBNP) is a method that reduces central venous pressure by redistributing the blood towards the lower body via the use of a vacuum chamber that is sealed at the iliac crest [1,2]. LBNP induces a reduction in cardiac preload that significantly lowers the stroke and end-diastolic volume in a linear relationship with the degree of negative pressure applied. LBNP has been shown to be a non-invasive surrogate to study physiological responses to blood loss/haemorrhage, a leading cause of death in patients with trauma [3,4]. The pressure gradient created by LBNP causes a migration of intravascular fluid to the lower body’s extravascular compartment, causing a rise in hemoconcentration. This leads to increases in blood viscosity and plasma protein concentrations, and elevates interactions between procoagulant cellular factors and coagulation factors, thus creating a procoagulant environment [5] which is seen in both sexes [6,7,8]. However, how seasonal variation in coagulation parameters occurs in both sexes and how they are influenced by LBNP have not yet well been researched.

Current knowledge about biological fluctuations on the seasonal variation of hemostatic parameters is mostly based on studies with a small sample size and/or the evidence is still limited [9]. We hypothesized that the baseline coagulation values present a more procoagulant pattern during the cold season. This hypothesis is based on previous observations that in winter months there is an increase in venous thromboembolism (VTE) [10,11], rise in cardiac infarctions [12,13,14] and/or general cardiovascular mortality [15]. In addition, it has been reported that there is an increase in factors that promote coagulation (e.g., fibrinogen, plasma viscosity) [16]. However, to our knowledge no study has examined how baseline coagulation values differ across the sexes or are influenced by central hypovolemia, which is commonly simulated in the laboratory via LBNP or in daily life via orthostatic loading (standing up for a supine position), during warmer and colder months.

It is important to study sex differences as the haemostatic system of women differs from that of men in various aspects. For example, women as compared to men have lower median levels of the anticoagulant protein S (PS) and thrombelastometry (TEM) measurements show an earlier onset and an elevated coagulation velocity [17,18]. We have recently reported differences in baseline coagulation parameters between men and women and also how central hypovolemia, induced by LBNP, affects coagulation responses [3]. We observed that at baseline, women were hypercoagulable compared with men, as evidenced by their shorter “Lag times” and higher thrombin peaks and by shorter “Coagulation times” and ‘Clot formation times’. Moreover, men were more susceptible to LBNP, as reflected in their elevated FVIII levels and decreased “Lag times” following LBNP. LBNP-induced coagulation activation was also not accompanied by endothelial activation. It appears that application of LBNP might be a useful future tool to identify individuals with an elevated risk for thrombosis across the seasons in subjects with or without history of thrombosis. The results from our study could have clinical implications. Patients with an elevated risk for thrombosis in different seasons might be identifiable. An anticoagulant treatment of these singled-out patients might be essential to prevent future thrombotic events, especially during certain times of the year.

## 2. Experimental Section

An approval was obtained from the Ethics Committee of the Medical University of Graz, before starting the study. Subjects also provided written and informed consent.

### 2.1. Subjects

Inclusion criteria were: males and females of age 18–35 years and height 160–180 cm. The criteria for exclusion were: endurance athletes, smokers, pregnant subjects, and subjects with/having a family history of cardiovascular diseases, thrombosis, coagulation disorders, orthostatic intolerance, or intake of any medication known to affect the coagulation system. Women on oral contraceptive pills were not excluded. Each person carried out the measurements in the cold (November–April) and warm months (May–October) [19].

### 2.2. Sample Size

The number of participants required to show statistical significance was based on previously published studies in which coagulation parameters were assessed during central hypovolemia [3,20,21]. An error probability (α) of 0.05, power (1 − β) of 0.80 and an average effect size (*d*) of 0.8 were used to calculate the sample size [21].

### 2.3. Study and LBNP Protocol

Prior to the experiments, the subjects were advised to refrain from coffee and alcohol for up to 24 h. The testing sessions were performed at 9–11 am in a partially darkened quiet room. Room temperature was maintained at 23–24 °C and humidity at 55–60%.

Each experiment commenced with the subject in a supine position for 30 min, during which time the electrodes for cardiovascular monitoring were placed on the subjects [22]. A graded LBNP protocol was used: suction started at −10 mmHg, and then was increased in five-minute intervals by −10 mmHg, until a maximum suction of −40 mmHg (a level at which the fluid shifts and central hypovolemia are similar to upright standing and most subjects are able to tolerate without developing presyncopal signs and symptoms) [23,24]. Finally, a ten-minute recovery period was allowed. Development of any presyncopal signs and symptoms such as blurring of vision, nausea, abdominal discomfort or sudden decreases in heart rate and blood pressure [25,26] led to termination of the experiments. However, none of the participants experienced any presyncopal signs or symptoms during LBNP application in summer and winter months.

### 2.4. Blood Sampling

Blood sampling occurred from a vein in the antecubital fossa at: baseline, end of −40 mmHg LBNP and at 10 min post-LBNP. Blood was collected into precitrated Vacuette^®^ containers (Greiner Bio-one GmbH, Kremsmünster, Austria) and within three hours of sampling the following were analyzed: TEM, whole blood (WB), aggregation of platelets. Then a platelet poor plasma (PPP) sample was obtained by centrifuging a part of the blood sample at room temperature at 1200 g for 15 min. PPP was used to measure conventional clotting times, pro- and anticoagulant proteins, thrombin generation curves and markers of thrombin generation as described in detail elsewhere [20]. Briefly, the activated partial thromboplastin time (aPTT), prothrombin times (PTs), plasma levels of protein C (PC), protein S (PS), and FII, FVII and FVIII were measured (Boehringer Mannheim/Hitachi 917, Mannheim, Germany). Prothrombin fragments 1+2 (F1+2), plasma thrombin–antithrombin complexes (TATs), Tissue-Plasminogen Activator (tPA) concentration and Tissue factor (TF) were measured using commercially available ELISA kits. Calibrated automated thrombography (CAT) was carried out to monitor thrombin generation curves (Thrombinoscope BV, Maastricht, Netherlands). CAT was used to measure the lag time—lag time preceding the thrombin burst, ETP: endogenous thrombin potential, Peak: peak height, ttPeak: time to peak, peak rate of thrombin generation (peak thrombin/(peak time − lag time)) (VELINDEX), and StartTail: the time point at which free thrombin disappears. The presence of low amounts of tissue factor (TF) was used to detect thrombin formation. In addition, the tissue factor-triggered TEM assay (thrombelastometer) provided the following measurements: Clot Formation Time (CFT), Coagulation Time (CT): Maximum Clot Firmness (MCF) and Alpha angle. Finally, Hematocrit (Hct), Hemoglobin (Hb), mean corpuscular haemoglobin concentration (MCHC), mean corpuscular haemoglobin (MCH), mean corpuscular volume (MCV) and blood cell counts were determined using an Automated Hematology Analyzer (Sysmex, Illinois, USA). 

### 2.5. Statistics

All the data are presented as mean ± SD. A Shapiro–Wilk W test was performed to test for normal distribution of all variables. Most variables were normally distributed. As the main objective of the study was to assess whether there is a difference in coagulation across two seasons, at rest and during LBNP application, the difference in the effect of LBNP on the subjects’ coagulation was measured at three points (baseline, LBNP at −40 mmHg, and after 10-min recovery phase) across two timeframes (cold and warm months). This comparison was carried out using a repeated measures ANOVA, and included the factors seasons and interaction between seasons and time (baseline, LBNP at −40 mmHg, recovery); Dunn’s post-test was then carried out. The Mann Whitney U-test was applied to analyze the seasonal variation in the baseline coagulation values. GraphPad 7.05 was used to for statistical calculations and data analyses. Statistical significance was established at *p* < 0.05. * *p* ≤ 0.05, ** *p* ≤ 0.01, *** *p* ≤ 0.001.

## 3. Results

Seven men (25.0 ± 3.6 years, 79.7 ± 7.8 kg body weight, 182.4 ± 3.3 cm height, and 23.8 ± 2.3 kg/m^2^ BMI) and six women (25.0 ± 2.4 years, 61.0 ± 8.4 kg body weight, 167 ± 4.7 cm height, and 21.8 ± 2.4 kg/m^2^ BMI) participated in this study. All participants completed the study protocol (that is, no presyncopal event occurred during LBNP application).

### 3.1. LBNP Effects on Standard Coagulation Parameters

**Figure 1:**
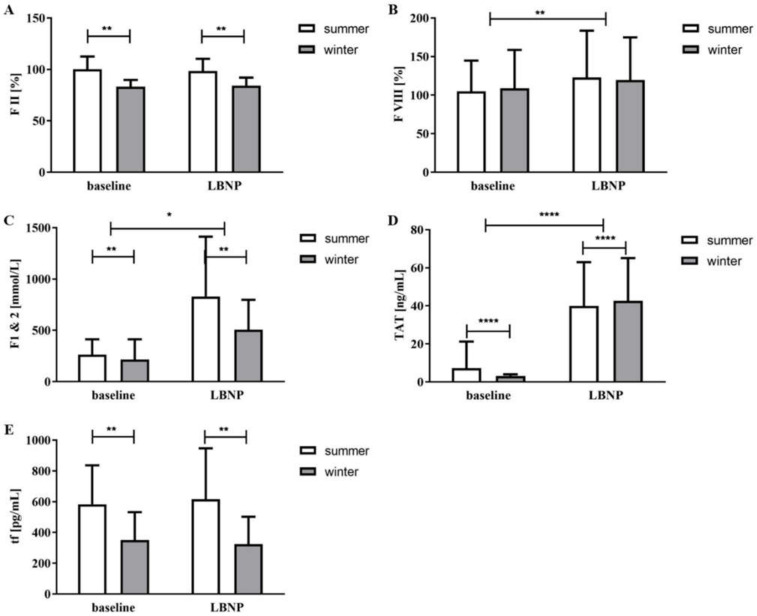
Coagulation variables during summer and winter at baseline, during LBNP and recovery: (**A**) Factor II; (**B**): Factor VIII; (**C**): F1 + F2 levels; (**D**): thrombin/antithrombin complex (TAT ); (**E**) tissue factor. Data are expressed as mean ± SD. * *p* ≤ 0.05, ** *p* ≤ 0.01, **** *p* ≤ 0.0001.

In summer, FII baseline levels were higher, both at baseline as well as during LBNP (Figure 1A). Other parameters such as PT, aPTT, FVII, VIII, PC and PS were not different across the seasons. LBNP suction caused a shortening of aPTT during summer (*p* = 0.018) and PT during winter (*p* = 0.028) (Table 1). In both seasons, LBNP application led to significant increases in FVIII (summer *p* = 0.021, winter *p* = 0.0053) (Table 1, Figure 1B). LBNP suction had no effect on Factor II, VII, PC and PS.

### 3.2. LBNP Effects on Blood Cell Count, Haematocrit, Haemoglobin, MCHC, MCH and MCV

The respective baseline levels of RBC, Hct, Hb, WBC, MCH, MCHC, and MCV were similar across seasons. Hematocrit decreased during LBNP followed by a rise in the recovery phase in both seasons (summer *p* ≤ 0.0001; winter *p* = 0.0039). A similar pattern of changes was also seen for WBC, RBC and Hb. LBNP application did not, however, affect MCHC, MCH and MCV. LBNP application did not affect platelet levels in both seasons (Table 1).

### 3.3. LBNP Effects on Thrombin Generation Markers

F1+2 and TAT levels were greater in the warmer season and also showed higher responses to LBNP application (Table 1).

### 3.4. Thrombin Generation Using CAT and TEM

The baseline measurements of thrombin generation using CAT (endogenous thrombin potential (ETP), lag time (LT), Peak, ttPeak, Velocity Index, StartTail) and using thrombelastometry (MCF, CT, CFT and alpha angle) did not show a seasonal variation.

### 3.5. CAT Measurements during LBNP

In the warmer months, LBNP application led to decreases in LT (*p* = 0.0028) and ttPeak (*p* = 0.0025) (with an eventual increase in levels during recovery) whilst increases in Peak (*p* = 0.014) and Velocity Index (*p* = 0.036) were seen (Table 1). On the other hand, in colder months, LBNP application did not affect any of the CAT measured variables. Neither StartTail nor ETP were affected by seasons (Table 1).

### 3.6. TEM Measurements during LBNP

CT was reduced in both seasons (summer *p* = 0.023, winter *p* = 0.042,). All other TEM-measured values—with the exception of Alpha, which increased in winter *p* = 0.035)—were not influenced by LBNP (Table 1).

Finally, statistical analysis of seasonal dependency using the means of the ANOVA only showed significant seasonal differences in F1+2 (*p* = 0.0023, Figure 1C) and TAT (*p* = 0.0001, Figure 1D). This is in contrast to markers measured via CAT and TEM, which did not show any significant seasonal variation.

### 3.7. Endothelial Activation during LBNP

In the summer months tissue factor levels were greater (Figure 1E). t-PA was, however, not affected by seasons. TF and t-PA were both not influenced by LBNP application. The absolute values of TF during LBNP application were, however, significantly higher in summer (Figure 1E).

## 4. Discussion

In young healthy adults, we observed a higher baseline level of FII, TF, F1+2, and TAT during summer. We also found a more pronounced LBNP-induced procoagulative effect, especially on CAT parameters (LT, Peak, ttPeak, Velindex) during summer (Table 1). On the other hand, the coagulation-related parameters did not show higher basal levels during winter. The only variables that changed significantly in response to LBNP, exclusively in winter, were PT and Alpha. Finally, LBNP affected—regardless of the season—FVIII, WBC, RBC, Hct, Hb, F1+2, TAT, and CT. As the lag time represents the delay of initial thrombin generation and ttPeak to the time it takes the blood to reach the point of maximum velocity of thrombin generation (Peak) [27], and thrombin generation reflects the haemostatic/prothrombotic state of the blood [28], our observations suggest that the coagulation system shows a greater response to the LBNP-induced central hypovolemia in warmer months. For instance, shorter ttPeaks in summer months during LBNP suggests a faster maximum thrombin formation thus implying a hypercoagulable state compared with baseline. To our knowledge, the seasonality of the coagulation response to either LBNP application or during orthostatic loading has not been previously studied.

As outlined above, higher basal concentrations of FII, TF, and F1+2 suggest greater coagulability during the summer, an observation that has not been reported previously but could have a significant clinical application. For example, venous thrombosis development is promoted by higher prothrombin (FII) levels [29,30]. This is believed to be partly caused by changes in the structure of the resulting fibrin clots [30]. In addition, all thrombin generation markers (F1+2, TAT, and D-dimer) [31] have been reported to be elevated in patients with thrombosis [32]. Indeed, in a cohort of cancer patients (*n* = 821) it was observed that elevated levels of F1+2 and D-dimer were associated with the occurrence of thromboembolic incidents [33]. For instance, a 15.2% cumulative probability of VTE was found after 6 months in the group with elevated F1+2 and D-dimer levels, as compared to 5.0% in the rest of the patients [33]. Finally, tissue factor was greatly elevated in warmer months (583.3 ± 241.9 pg/mL vs. colder months (351.1 ± 165.9 pg/mL). TF is known as an initiator of coagulation via the extrinsic pathway when it comes in contact with Factor VII [34]. Since the concentration of TF needed to initiate the formation of thrombin depends on the amount of anti- and procoagulant factors [5] it is now considered a mediator between greater concentrations of procoagulant factors and the VTE risk. TF levels have been associated with thrombotic incidents [8] and with myocardial infarction [6,35]. TF expression by monocytes and endothelial tissue can be increased by inflammatory and immunological processes [7,36].

Hematocrit, haemoglobin, red blood cells and white blood cells demonstrate similar responses across seasons. An LBNP-induced shift of fluid from an intracellular to extracellular space could have led to the RBC and WBC changes observed, as has been previously reported by Zaar et al. The platelet count was not significantly altered by LBNP application [37]. This is in contrast to some studies that have reported elevations in platelets following LBNP applications [37,38,39]. We did not see such changes across the seasons or during LBNP application in each season, which could potentially be attributed to the lower suction level (−40 mmHg) used in our study.

While FVIII plasma levels have been shown to be increased significantly by LBNP in females and males [3,40,41], we are not aware of any study that has reported how FVIII levels change with LBNP application during different seasons. In our study, we observed LBNP-induced increases in factor VIII in both seasons. LBNP-associated Factor VIII could be explained by the adrenergic activation triggered by LBNP [36]. Adrenergic physiological stress reactions are also known to occur during pregnancy, in liver diseases or with increasing age and these conditions, in turn, are known to raise the concentration of Factor VIII. The occurrence of VTE increases with higher levels of FVIII in a dose-dependent relationship [42]. Additionally, blood viscosity, haematocrit, platelet count, and plasminogen activator inhibitor 1 [16] and D-dimer levels [43] have been reported to be elevated in colder months. In winter there is an increase in admissions and mortality related to myocardial infarction [14,44,45] and greater incidence of VTE [10,11]. In addition, during winter, a more procoagulant state in the blood—as suggested by the increased levels of fibrinogen [12,16,46,47] and Factor VII [13,46]—is seen.

Our results to some extent contrast with those that are obtained from other studies, in which coagulation during different seasons was reported. There may be several reasons for this: Firstly, our study was carried out in healthy young subjects. Most of the studies, even with a relatively big sample size (*n* = 2325), reported coagulation changes, which are most pronounced in elderly subjects [47]. Only Fröhlich et al. included young and healthy subjects (*n* = 16) in their study in which they assessed if increased synthesis of acute-phase proteins such as fibrinogen due to infections in winter may be associated with greater morbidity and mortality from cardiovascular diseases [16]. Their results were somewhat inconclusive [16]. Other studies also examined coagulation changes across seasons but these studies mainly included subjects of middle age and older persons. Van der Bom et al. described an age-dependent pattern of coagulation parameters, with the seasonal associated rise in fibrinogen during winter being mostly observable in the elderly [47]. As our subjects were relatively young, it is difficult to compare seasonal variations in coagulation with these three studies. Moreover, we did not measure fibrinogen, which has been well-established as a risk factor for VTE in a concentration dependent manner [48]. Secondly, we assessed the effects of central hypovolemia induced by LBNP on coagulation parameters while other studies compared the coagulation system without any perturbation. When we tried to compare our baseline values with those of other studies, it was difficult as there is relative lack of literature related to coagulation alterations during the seasons. Thirdly, room and environmental temperature conditions during testing could have contributed to some of the inconsistencies between our results and other studies. For example, Stout and Crawford reported a negative association between body and environmental temperature and fibrinogen levels [12]. While the data from Stout ad Crawford were collected from patients places of living, Van der Bom et al. (1997) studied fibrinogen levels in patients who underwent blood sampling at a research facility (with fewer temperature fluctuations) reported no such correlations between fibrinogen levels and outdoor temperatures [47,49]. The participants in our study were in a temperature- and humidity-controlled laboratory during both seasons. However, to what extent external/outside/environmental temperature could have affected our results is difficult to ascertain from our study.

Baseline levels of endothelial activation markers TF and tPA levels were the same in warm and cold months and not significantly altered by LBNP. Thus, LBNP appears to be a suitable method not only to simulate central hypovolaemia but also to expose individuals to a procoagulant challenge without massive endothelial activation associated with a possible risk of inducing subsequent thrombosis in varying seasons. On the other hand, sit-to-stand tests or LBNP in combination with graded LBNP could potentially activate the endothelium and coagulation cascade with dire consequences [50].

An important limitation of this study is that the data shown here are pooled data from males and females and also do not take into account menstrual cycle phases or whether the females were taking oral contraceptives (OCs). While this could be a limitation, as the sex of the participants (Cvirn et al. 2019) [3] and the menstrual phases (Goswami et al. 2020) [51] are known to affect coagulation, we did not include these aspects in our data analysis, as assessment of differences across the sexes or phases of the menstrual cycle were not the aims of this study. Future studies should examine effects of seasons on coagulation parameters across the sexes of the participants and phases of the menstrual cycle.

## 5. Conclusions

In conclusion, our study shows a more procoagulant environment (greater prothrombin time and shorter ttpeak values, implying faster maximum thrombin formation and a stronger activation of the coagulation system in response to LBNP during summer (as reflected in their decreased aPTT and elevated FVIII, F1+2, and TAT levels)). As current knowledge of the seasonal dependency of the coagulation system is still limited, and mostly based on studies with a small number of subjects [9], our novel results provide further insight into the seasonal variability of the coagulation system, which could help to identify seasonal bias in future studies, better understanding of factors influencing the pathogenesis of coagulation-related incidents, and may have clinical implications in relation to those diseases. Finally, LBNP represents a mild but efficient stimulus to expose individuals to a procoagulant challenge. LBNP, therefore, appears to be superior to a stand test. While a simple sit-to-stand test might also have the potential to identify individuals with an increased risk of thrombosis, it is accompanied by an activation of both the endothelium and the coagulation cascade and might therefore not be appropriate in patients with higher risk of thrombosis. However, the present study shows that LBNP is a very mild but efficient coagulation stimulus and, thus, might be a suitable tool to screen for thrombosis.

Future studies should assess larger cohorts of young and older persons and, in males and females, how resting coagulation parameters are influenced by seasons, external temperature, room temperature or in the presence/absence of any underlying inflammation/disease. Understanding changes in coagulation during changes in posture from lying to standing up is important as standing up has been associated with a procoagulant status [52]. Finally, as plasma aldosterone and plasma renin activity (PRA) show increases of up to 59 and 17%, respectively, from summer to winter months [53], future research should assess how these hormonal changes in volume-regulating hormones [54] correlate with coagulation changes across seasons.

## Figures and Tables

**Table 1:** An overview of all the coagulation and hematological values at rest, during Lower body negative pressure (LBNP) and recovery. Significant p-values are bold.

Season	Variable	Baseline	LBNP	Recovery	*p*-Value (ANOVA)
Summer	aPTT (s)	34.67 ± 2.78	32.30 ± 3.09	33.25 ± 4.47	**0.018**
	PT (%)	103.92 ± 13.10	107.23 ± 13.73	104.08 ± 15.31	0.17
	FII (%)	103.31 ± 15.72	102.23 ± 17.14	103.67 ± 15.50	0.91
	FVII (%)	105.77 ± 35.15	107.85 ± 36.79	107.42 ± 37.44	0.61
	F VIII (%)	105.00 ± 36.63	122.62 ± 55.92	126.42 ± 64.26	**0.021**
	PC (%)	97.23 ± 31.31	101.00 ± 27.83	98.50 ± 31.77	0.56
	PS (%)	85.85 ± 16.26	85.15 ± 22.70	91.92 ± 20.88	0.35
	WBC (103/µL)	5.11 ± 0.98	4.78 ± 1.11	5.43 ± 1.40	**0.0001**
	Plt (103/mL)	203.15 ± 55.14	198.40 ± 54.00	208.58 ± 56.35	0.063
	RBC (106/mL)	4.33 ± 0.40	4.26 ± 0.40	4.38 ± 0.40	**0.0001**
	Hct (%)	37.12 ± 3.31	36.52 ± 3.36	37.44 ± 3.31	<**0.0001**
	Hb (g/dL)	12.72 ± 1.38	12.58 ± 1.39	12.89 ± 1.46	<**0.0001**
	MCHC (g/dL)	34.24 ± 1.12	34.40 ± 1.17	34.36 ± 1.24	0.35
	MCH (pg)	29.37 ± 1.38	29.53 ± 1.33	29.38 ± 1.47	0.26
	MCV (fL)	85.78 ± 2.77	85.77 ± 2.69	85.50 ± 2.70	0.94
	F1+2 (pmol/L)	263.7 ± 142.6	776.3 ± 567.3	1078.4 ± 837.4	**0.0027**
	TAT (ng/mL)	7.26 ± 13.36	37.21 ± 23.23	42.02 ± 23.62	**0.0004**
	TF (pg/mL)	583.3 ± 241.9	593.7 ± 314.2	575.3 ± 169.0	0.63
	tPA (ng/mL)	2.12 ± 1.96	1.45 ± 1.09	1.58 ± 0.85	0.25
	LT (min)	2.36 ± 0.30	2.31 ± 0.24	2.46 ± 0.27	**0.028**
	ETP (nM min)	1487.5 ± 449.3	1529.0 ± 417.3	1501.9 ± 484.7	0.20
	Peak (nM)	273.30 ± 94.39	287.03 ± 77.09	263.55 ± 101.01	**0.014**
	ttPeak (min)	5.61 ± 0.83	5.38 ± 0.57	5.92 ± 0.97	**0.0025**
	VelIndex (nm/min)	91.54 ± 43.94	97.38 ± 35.92	85.53 ± 46.76	**0.036**
	StartTail (min)	21.18 ± 1.73	21.29 ± 1.50	21.82 ± 1.79	0.27
	CT (s)	199.38 ± 46.33	172.92 ± 33.57	188.83 ± 32.53	**0.023**
	CFT (s)	151.23 ± 74.70	121.23 ± 38.37	142.67 ± 59.64	0.27
	MCF (mm)	56.77 ± 6.73	58.00 ± 5.72	56.67 ± 5.93	0.49
	Alpha (°)	63.00 ± 9.58	66.69 ± 6.19	64.17 ± 8.91	0.27
Winter	aPTT (s)	35.62 ± 6.05	33.69 ± 4.62	36.98 ± 16.16	0.71
	PT (%)	103.85 ± 11.86	101.62 ± 10.88	106.00 ± 11.18	**0.028**
	FII (%)	85.69 ± 10.65	84.25 ± 7.52	85.17 ± 5.26	0.46
	FVII (%)	107.62 ± 22.75	108.17 ± 23.30	109.00 ± 24.17	0.69
	F VIII (%)	107.77 ± 45.85	119.58 ± 52.92	124.75 ± 54.96	**0.0053**
	PC (%)	94.15 ± 17.42	95.58 ± 15.11	95.08 ± 19.41	0.98
	PS (%)	83.23 ± 23.48	88.00 ± 25.73	83.33 ± 16.04	0.61
	WBC (103/µL)	4.76 ± 0.77	4.23 ± 0.59	4.93 ± 0.79	<**0.0001**
	Plt (103/mL)	192.54 ± 42.19	195.08 ± 38.50	195.62 ± 36.90	0.64
	RBC (106/mL)	4.27 ± 0.33	4.25 ± 0.37	4.33 ± 0.35	**0.0036**
	Hct (%)	36.72 ± 2.56	36.54 ± 2.91	37.26 ± 2.64	**0.0039**
	Hb (g/dL)	12.69 ± 1.07	12.64 ± 1.22	12.89 ± 1.15	**0.0088**
	MCHC (g/dL)	34.54 ± 1.01	34.54 ± 1.04	34.56 ± 1.00	0.97
	MCH (pg)	29.73 ± 0.99	29.74 ± 0.97	29.76 ± 1.00	0.91
	MCV (fL)	86.09 ± 2.43	86.10 ± 2.33	86.12 ± 2.27	0.98
	F1+2 (pmol/L)	212.3 ± 27.80	546.2 ± 99.37	584.3 ± 108.7	**0.036**
	TAT (ng/mL)	3.30 ± 0.32	41.77 ± 8.81	41.19 ± 9.96	**0.004**
	TF (pg/mL)	351.1 ± 165.9	324.2 ± 170.4	377.92 ± 137.9	0.49
	tPA (ng/mL)	1.88 ± 1.66	1.71 ± 0.99	1.59 ± 0.85	0.8
	LT (min)	2.13 ± 0.10	2.22 ± 0.11	2.27 ± 0.14	0.184
	ETP (nM min)	1688 ± 214	1645 ± 210	1645 ± 197	0.347
	Peak (nM)	312.80 ± 43.15	304.50 ± 42.68	297.30 ± 38.44	0.315
	ttPeak (min)	5.10 ± 0.31	5.19 ± 0.26	5.35 ± 0.28	0.239
	VelIndex (nm/min)	116.60 ± 24.64	110.90 ± 21.30	103.20 ± 19.57	0.079
	StartTail (min)	21.46 ± 0.43	21.55 ± 0.39	21.44 ± 0.41	0.906
	CT (s)	155.60 ± 13.11	144.40 ± 13.00	148.40 ± 13.48	0.076
	CFT (s)	121.70 ± 15.53	107.30 ± 20.56	99.86 ± 11.04	0.122
	MCF (mm)	60.86 ± 2.06	62.00 ± 1.98	62.00 ± 2.01	0.500
	Alpha (°)	66.14 ± 2.49	69.57 ± 3.12	70.00 ± 2.05	**0.035**

*Legend:* CAT: calibrated automated thrombography; CFT: clot formation time; CT: coagulation time; ETP: endogenous thrombin potential; F 1 + 2: prothrombin fragment 1+2; Hct: haematocrit; Hb: haemoglobin; LT: lag time; MCF: maximum clot firmness; MCH: mean corpuscular haemoglobin; MCHC: mean corpuscular haemoglobin concentration; MCV: mean corpuscular volume; OC: oral contraceptives; PC: protein C; Plt: platelet count; PS: protein S; PT: prothrombin times; RBC: red blood cell counts; TAT: thrombin/antithrombin complex; TEM: thrombelastometry; TF: tissue factor; t-PA: tissue-plasminogen activator; WB: whole blood; WBC: white blood cell counts. All data are mean ± SD.

## Abbreviations

aPTT	activated partial thromboplastin time
CAT	calibrated automated thrombography
CFT	clot formation time
CT	coagulation time
ETP	endogenous thrombin potential
F1+2	prothrombin fragment 1+2
Hct	Haematocrit
Hb	Haemoglobin
LBNP	lower body negative pressure
LT	Lag time
MCF	maximum clot firmness
MCH	mean corpuscular haemoglobin
MCHC	mean corpuscular haemoglobin concentration
MCV	mean corpuscular volume
OC	oral contraceptives
PC	protein C
Plt	platelet count
PS	protein S
PT	prothrombin times
PPP	platelet poor plasma
RBC	red blood cell counts
SD	standard deviation
TAT	thrombin/antithrombin complex
TEM	thrombelastometry
TF	tissue factor
t-PA	tissue-plasminogen activator
VTE	venous thromboembolism
WB	whole blood
WBC	white blood cell counts.
